# Determining toxins and harmful contaminants in starfish for future application as organic fertilizer and animal feed

**DOI:** 10.1007/s11356-025-36430-3

**Published:** 2025-05-07

**Authors:** Marta Turull, Belén Budiño, Philippe Savarino, Pascal Gerbaux, Maria Rambla-Alegre, Santiago Cabaleiro, Sergi Díez

**Affiliations:** 1 Environmental Chemistry Department, Institute of Environmental Assessment and Water Research (IDAEA-CSIC), E- 08034 Barcelona, Spain; 2 Technology Center of the Aquaculture Cluster (CETGA), Punta de Couso, s/n Aguiño, A Coruña, Ribeira E- 15965 Spain; 3 Organic Synthesis & Mass Spectrometry Lab, University of Mons (UMONS), 23 Place du Parc, Mons, B- 7000 Belgium; 4 IRTA, Marine and Continental Waters, Ctra. Poble Nou, km. 5.5, E- 43540 La Ràpita, Spain

**Keywords:** Marthasterias glacialis, *Asterias rubens*, Galician coast, Metals, Organic pollutants

## Abstract

Starfish play a crucial role as extraordinary predators in marine ecosystems. They often prey on bivalves, which serve as primary economic resources in certain regions, making their management a top priority. As a result, cleaning operations have been conducted to remove starfish from the coasts, leading to the accumulation of significant waste. Within the context of the circular economy, there is potential for valorizing starfish, transforming them from waste products into valuable resources. However, before doing so, it is essential to analyze the contaminants that starfish can accumulate. To address this, a study was conducted to analyze pollutants in two species of starfish: *Marthasterias glacialis* and *Asterias rubens*, collected from the Galician Spanish shores. The goal was to assess their suitability for use as animal feed or fertilizer in agriculture. The study employed optimized methodologies to analyze trace elements, saponins, marine toxins and persistent organic pollutants (POPs), including polychlorinated biphenyls (PCBs), organochlorine pesticides (OCPs) and polycyclic aromatic hydrocarbons (PAHs). The validated protocols for determining these contaminants in starfish showed an acceptable performance in detection, showing the quality parameters of the analytical analysis (recoveries, precision, limit of detection (LOD) and quantification (LOQ)). Results indicated that concentrations of the legislated contaminants in the starfish samples from the Galician shores did not exceed the current EU legislation standards. Consequently, in terms of toxicology, starfish matrix could be considered a suitable raw material for the use in animal feed or as crop fertilizer.

**Supplementary information:**

The online version contains supplementary material available at 10.1007/s11356-025-36430-3.

## Introduction

Starfish are impressive predators in marine ecosystems. These echinoderms use their tube feet to open the shells of bivalves, such as clams and oysters. Some species, like the crown-of-thorns starfish, are known for their voracious appetite for coral polyps, posing significant threats to coral reef health in several places such as the Gulf of California and the Great Barrier Reef (Castro-Sanguino et al. 2023; Matthews et al. 2024). Their predatory behavior not only regulates prey populations but also indirectly influences the community structure of the ocean floor and the economy along their region’s coastline. Over the past decade, there has been a notable proliferation of starfish, primarily *Asterias rubens* and *Marthasterias glacialis*, detected along the shores of Galicia. These starfish are now considered the primary predators of bivalves. Galicia, located in the far west of Spain and bordered by both the Atlantic Ocean and the Cantabrian Sea, holds a significant position as Europe’s most substantial mussel producer and ranks second globally, just after China. Annually, Galicia produces 250,000 tons of mussels, utilizing around 3300 rafts (Caballero Miguez et al. 2009). Consequently, this situation poses a significant challenge to the exploitation of mussels, clams and razon clams in the region. Cleaning operations to mitigate the proliferation of starfish along the shores have been conducted since 2002 (La voz de Galicia 2006), resulting in the collection of approximately 40 tons of animals per year. After the extraction from the sea, the animals are either transported to other locations away from seafood banks or treated as a waste, managed by a specialized company. Unfortunately, there is currently no recognized potential use of this waste.

Given the challenges associated with the proliferation of starfish along the Galician coasts, it is important to explore the potential of utilizing starfish as a value-added product rather than considering them as waste. Previous studies have demonstrated that starfish gonads exhibit an elevated nutritional profile, comparable to that of mussels, with high protein content (Taboada et al. 2008). However, it is worth noting that starfish have been found to contain toxins that are harmful to mammals upon oral administration. Paralytic toxins (Lin and Hwang 2000), gonyautoxins (Ben-Gigirey et al. 2020), neurotoxins (Dean et al., 2021), and asterosaponins (Mendoza-Porras et al. 2023) have been detected in various species of starfish.

Currently, mainly due to toxins, consumption by humans or animals is not viable without adequate treatment. Several studies have shown that the toxicity of starfish varies depending on the species and geographical location. However, these toxic substances can be neutralized through heat treatments at temperatures of 56–60 °C, exposure to extreme pH levels (3 to 10), or repetitive cycles of freeze–thaw at − 20 °C (Grotendorst and Hessinger 2000; Kanagarajan et al. 2008). Therefore, exploring appropriate processing methods could potentially mitigate the toxicity issues associated with starfish, making them a viable resource for various applications, thus fitting within the circular economy framework.

In this context, the main objective of the study is to evaluate the viability of using starfish in the future as a raw material for animal feed and/or fertilizer in agriculture. To achieve this goal, two interrelated objectives have first been outlined: (i) validate the analytical procedures to determine trace elements, persistent organic pollutants (POPs), marine toxins and saponins, since there are currently no standardized protocols for starfish matrices; and (ii) determine the concentration of these regulated contaminants to ensure that starfish waste does not surpass EU-mandated limit values.

## Materials and methods

### Starfish samples

For this study, multiple random specimens were subsampled to create a composite sample that was used for further analysis for both species of starfish. For *A. rubens*, samples were collected between February and November 2020, resulting in four composite samples (50 kg each) obtained in different months from Campelo, Illa de Arousa, Noia, and Campelo. In contrast, a single composite sample (200 kg) of *M. glacialis* was collected from different batches collected between July and August 2020 in Agüiño and Ribeira. For *A. rubens* and *M. glacialis*, four and one composite samples, respectively, were composed and used for further analysis by subsampling random specimens of each species. However, the results were determined through the analysis separated by species rather than by individual samples.

The starfish specimens were rinsed with seawater and sacrificed by thermal shock. Their entire bodies were then crushed using an industrial meat grinder. The homogenized samples were drained of excess water using an 80-μm nylon mesh, then divided into two portions and frozen at − 20 °C. One portion was used for the analysis of marine toxins and saponins and was analyzed directly in its wet weight form. The other portion was lyophilized (Telstar Technologies, SL, Spain) and subsequently stored at 4 °C until analysis of metals and POPs.

### Reagents and materials

Hydrogen peroxide (30%, Selectipur) was purchased from Fisher Scientific (UK, limited), and concentrated nitric acid (69%, EMSURE) was obtained from Merck (Darmstadt, Germany). Dichloromethane, ethanol, and ethyl acetate were acquired from Sigma-Aldrich (St. Louis, MO, USA). Acetone, silica gel, anhydrous sodium sulfate, and florisil were obtained from Merck (Darmstadt, Germany), and hexane was obtained from Honeywell (Germany). In addition, anhydrous sodium sulfate and florisil were activated at 150 °C for 24 h prior to use. High purity demineralized water (resistivity > 18.2 MΩ cm) was used for dilutions and preparation of test solutions (Millipore, Bedford, USA).

Certified reference materials of Hg (DORM- 3) and the other trace metals (DOLT- 5), including Al, As, Ca, Cd, Co, Cr, Cu, Fe, K, Mg, Mn, Na, Ni, P, Pb, Sr, and Zn, were purchased from the National Research Council of Canada (NRC, Halifax, NS, Canada).

For PCBs, standard solutions (PCB mixture including CB28, CB52, CB101, CB118, CB153, CB138 and CB180), the surrogates (CB65 and CB166) and the internal standard (CB30 and CB204) were acquired from Dr. Ehrenstorfer (Ausburg, Germany).

For OCPs, the standard solution (OCP mixture including Aldrin, α-BHC, β-BHC, δ-BHC, ϒ-BHC, 4,4′-DDD, 4,4′-DDE, 4,4′-DDT, dieldrin, endosulfan I, endosulfan II, endosulfan sulfate, endrin, endrin aldehyde, heptachlor, heptachlor epoxide and methoxychlor), the surrogates (2,4,5,6-tetrachloro-m-xylene and dibutyl chlorendate) and the internal standards (CB65 and CB166) were obtained from Dr. Ehrenstorfer (Ausburg, Germany).

For PAHs, the standard solution (PAH mix 9 including naphthalene, acenaphthylene, acenaphthene, fluorene, phenanthrene, anthracene, fluoranthene, pyrene, benzo[a]anthracene, chrysene, benzo[b]fluoranthene, benzo[k]fluoranthene, benzo[a]pyrene, indeno[1,2,3-cd]pyrene, dibenzo[a,h]anthracene and benzo[ghi]perylene), deuterated surrogates (naphthalene-d8, anthracene-d10, pyrene-d10, benzo[a]anthracene-d12 and perylene-d12) were purchased from Dr. Ehrenstorfer (Ausburg, Germany) and the internal standard (triphenylamine) were obtained from Merck (Darmstadt, Germany).

For the analysis of marine toxins, certified reference materials of lipophilic marine toxins (LMTs) of okadaic acid (OA), azaspiracid 1 (AZA-1), yessotoxin (YTX), and homoyessotoxin (hYTX) were purchased from Cifga (Lugo, Spain). Dinophysistoxin- 1 (DTX-1), dinophysistoxin- 2 (DTX-2), azaspiracid 2 (AZA-2), azaspiracid 3 (AZA-3), pectenotoxin-2 (PTX-2), 13-desmethylspirolide C (SPX-1), gymnodimine A (GYM-A) and pinnatoxin G (PnTX-G) were obtained from the National Research Council of Canada (NRC, Halifax, NS, Canada). Certified reference materials of amnesic shellfish poisoning (ASP) toxins including domoic acid (DA) was purchased from Cifga (Lugo, Spain). Certified reference material of paralytic shellfish poisoning (PSP) toxins including CRM- 00-dcGTX2&3, CRM- 00- C1&2, CRM- 00-dcSTX, CRM- 00-GTX2&3, CRM- 00-GTX5, CRM- 00-STX, CRM- 00-GTX1&4, CRM- 00-NEO and CRM- 00-dcNEO were purchased from the National Research Council (NRC, Halifax, NS, Canada) and CRM- 00-GTX6 was purchased from Cifga (Lugo, Spain). Acetonitrile and methanol hypergrade for LC–MS, MeOH gradient grade for HPLC and formic acid (98%) were purchased from Merck (Darmstadt, Germany). Ammonium bicarbonate and ammonium acetate (both elution additive for LC–MS), ammonium hydroxide (28% in water, v/v), ammonium formate for HPLC and sodium hydroxide (99%) were purchased from Sigma–Aldrich (Steinheim, Germany). Hydrochloric acid (37%, EMSURE) was purchased from Panreac Quimica (Barcelona, Spain). Formic acid was purchased from Merck (Darmstadt, Germany). Ammonium formate was from Fluka (Sigma-Aldrich, Spain). Glacial acetic acid reagent grade, Na_2_HPO_4_ analysis grade, sodium hydroxide reagent grade, ammonium acetate reagent grade and sodium chloride for analysis were purchased from Honeywell-Fluka (Schwerte, Germany), periodic acid analytical reagent AnalaR was from NORMAPUR (Sigma-Aldrich) and hydrogen peroxide solution (30%, v/v) reagent grade was from Panreac (Barcelona, Spain). SPE C18 sep-pack (3 mL, 500 mg) and SPE-Carboxylic Acid (3 mL, 500 mg) were obtained from JT Baker (Bellefonte, NJ, USA).

For saponins, methanol, n-hexane, chloroform, and isobutanol were purchased from Chem-Lab nv (Zedelgem, Belgium). Dichloromethane, dihydroxybenzoic acid. and N,N-dimethylalaniline were obtained from Sigma-Aldrich (St. Louis, MO, USA). LC-grade acetonitrile and formic acid were purchased from VWR Chemicals (Darmstadt, Germany). The internal standards for exact mass measurements (High Resolution Mass Spectrometry – HRMS), i.e. poly(ethylene glycol) PEG 600–1500 were obtained from ACROS Organics (Leicestershire, UK).

### Sample pre-treatment

#### Trace and major elements

For trace metal extraction, 0.2 g of powdered dried sample was accurately weighted and placed into PTEE microwave digestion vessels. Subsequently, 1 mL of milli-Q water, 2 mL of hydrogen peroxide, and 3 mL of concentrated nitric acid were added to perform the microwave digestion. To ensure quality control, digestion blanks and appropriate certified reference material (DOLT- 5) were included in each digestion run, following the same procedure. The microwave digestion program (Generic-Organic A 24HVT80 - 4*) consisted of the following steps: an initial ramp-up of 20 min from room temperature to 180 °C; followed by a 10-min hold at 180 °C, and finally, 20-min cooling period from 180 °C back to room temperature. After cooling, the digested samples were transferred to 50-mL vials and diluted with milli-Q water to 20 mL. The digested samples were then stored at 4 °C until analysis.

#### Persistent organic pollutants

Persistent organic pollutant compounds (POPs) were extracted from 1-g powdered dried sample using a Dionex ASE350 Accelerated Solvent Extractor (Dionex, USA). Before extraction, 25 µL of surrogates were added to the sample and allowed to equilibrate for at least one hour. The samples were placed into 10-mL cylindrical stainless steel extraction cells, which had been pre-cleaned with ethanol and acetone, and a cellulose filter specifically designed for ASE cells was placed at the bottom. The extraction was carried out using n-hexane/acetone (1:1, v/v) as the solvent, at a temperature of 100 °C, and a pressure of 1500 psi. The extraction process consisted of a 5-min heat-up time for the cell, followed by a 5-min static time, and 2 cycles of extraction. After extraction, the flush volume in the extraction cell was set to 60% of the cell volume and purged with nitrogen for 60 s. For PAH extraction, the temperature was increased to 140 °C.

Subsequently, the extracts were then concentrated to approximately 3 mL. Purification was carried out using different absorbents: (a) 2-g florisil adsorbent, eluted with 8-mL n-hexane for PCBs; (b) 3-g florisil adsorbent, eluted with 3 mL of hexane, followed by 5 mL of acetone:n-hexane (1:9, v/v), and finally, 5 mL of dichloromethane:n-hexane (1:3, v/v) for OCP; and (c) 2-g silica gel (3%, deactivated) and 1-g florisil, eluted with 8-mL dichloromethane:n-hexane (1:1, v/v) for PAH. In all cases, 100-mg anhydrous sodium sulfate were packed on top of the columns.

The extracts were then evaporated to near dryness under a nitrogen stream, and the residue was dissolved in 1 mL of ethyl acetate for PCBs and OCPs and 1 mL of n-hexane for PAHs. Prior to analysis, 25 µL of 1-ppm internal standard was added to each sample.

#### Saponins

The crushed samples (four pooled extracts for *A. rubens* and one pooled extract for *M. glacialis*) were ground using an analytical grinder (Analytical mill, IKA A 11 basic, Germany), and were dissolved in methanol while stirring for 16 h (~ 2 g in 35 mL). The suspension was then centrifuged at 4500 g for 10 min (Sigma 2 - 16P, Sigma, Osterode am Harz, Germany). The supernatant was then collected, and the extract diluted with water to reach a volume ration of 70/30 (methanol/water). This methanolic extract was partitioned (v/v) with n-hexane, chloroform, and dichloromethane to remove apolar compounds. The third aqueous phase is recovered and evaporated under vacuum using a rotary evaporator (IKA RV 10, IKA, Staufen, Germany) in a water bath (80 rpm, 50 °C) and the residue is brought to a volume of 25 mL in order to carry out a fourth liquid/liquid extraction (v/v) with HPLC isobutanol to recover the saponins in the organic phase. This phase is then washed twice with Milli-Q water to purify the extract from the residual salts and impurities. The organic phase, containing saponins, is evaporated under vacuum to obtain a purified powder.

#### Marine toxins

Marine toxins were extracted using three different methods depending on the nature of toxins.

For lipophilic marine toxin (LMT) extraction, samples were processed following the procedure described in the EU-Harmonised Standard Operating Procedure for determination of LMTs in mollusks by LC–MS/MS, as published by the EU-RL-MB (EURLMB 2011). 1 g of the shellfish tissue homogenate was weighted into a centrifuge tube, to which 3 mL of methanol was added. The sample was then vortex mixed for 3 min at 2500 rpm for thorough homogenization. After centrifugation (2000 rpm × 5 min) at 20 °C, the supernatant was transferred to a 10-mL volumetric flash. The extraction process was repeated with another 3 mL of methanol, and the supernatants from both extractions were combined to reach a final volume of 10 mL with methanol. The combined extract was then filtered through 0.2-μm PTFE filter into an amber HPLC vial, appropriately labeled. Total OA, DTX-2 and DTX-1 were detected following hydrolysis of the crude extract. For the hydrolysis, 1.25 mL of crude extract was transferred to a 2-mL amber vial, to which 125 µL of NaOH 2.5 N was added. After vortex homogenization, the sample was incubated in a thermoblock at 76 °C for 40 min. Following cooling to room temperature, 125 µL of HCl 2.5 N was added, vortex homogenized and filtered through a 0.2-µm PTFE filter into an amber HPLC vial.

For amnesic shellfish poisoning (ASP) toxins, samples were extracted according to the procedure described in the EU-Harmonised Standard Operating Procedure for determination of domoic acid in shellfish and finfish by RP-HPLC using UV detection (EURLMB 2008). 4 g of shellfish tissue homogenate was weighted into a centrifuge tube, and 16 mL of methanol:water (50:50, v/v) was added. The samples were vortex mixed for 3 min (2500 rpm) and then centrifuged (4500 rpm for 20 min). The supernatant was recovered in a 50-mL falcon tube and subjected to a second centrifugation at 4500 rpm for 20 min. The resulting supernatant was pooled, filtered through a 0.22-μm nylon filter into an amber HPLC vial, and injected into LC-UV.

For paralytic shellfish poisoning (PSP) toxins, the extraction was based on the UNE-EN 14526:2017 method (UNE 2017), which involved acetic acid extraction followed by clean-up with SPE C18 cartridge extraction, periodate oxidation, and analysis by HPLC-FLD. If any toxin was detected, peroxide oxidation and/or fractionation (F1, F2, F3) were conducted using carboxylic ion exchange SPE cartridges with periodate oxidation, and the obtained extracts were injected into HPLC-FLD.

### Instrumental analysis and quality assurance

#### Trace elements

The analysis of Hg was performed without previous extraction in dry weight using a Direct Mercury Analysis System (DMA- 80). The major elements (Al, Ca, Fe, K, Mg, Na, P and Sr) were analyzed on an inductively coupled plasma–optical emission spectrometer (Thermo Scientific iCAP 6500 Radial, ICP-OES), and trace elements (As, Cd, Co, Cr, Cu, Mn, Ni, Pb and Zn) were analyzed on an inductively coupled plasma–mass spectrometry (Thermo Scientific, XSeries 2 ICP-MS). Each sample (four pools of *A. rubens* and one of *M. glacialis*) was analyzed in triplicate. Recoveries were calculated by analyzing three reference materials as regular samples and comparing the obtained results with their theoretical reference values to determine the recovery of each element under study, as follows:

1 $$\text{Recovery }\left(\%\right)=\left(\frac{Observed\;value}{\text{Certified value}}\right)x100$$

#### Persistent organic pollutants

The purified extracts for the analysis of PCBs (including CB28, CB52, CB101, CB118, CB153, CB138 and CB180) and OCPs (including α-HCH, β-HCH, ɣ-HCH, δ-HCH, heptachlor, heptachlor epoxide, aldrin dieldrin, endrin, endrin aldehyde, endosulfan I, endosulfan II, endosulfan sulfate, 4,4′-DDE, 4–4′-DDD, 4–4′-DDT and metoxichlor) were analyzed by a gas chromatography using a Thermo Fisher Trace GC Ultra and ^63^Ni electron capture detector (GC-ECD). A CP-Sil 8 CB (Agilent Chrompack CP7482) capillary column, comprising 5% phenyl groups in dimethylpolysiloxane, with dimensions of 50 m × 0.25 mm i.d. × 0.25-µm film thickness, was used. For PCB analysis, the chromatographic conditions were as follows: the initial column temperature was held at 70 °C for 2 min, then increased to 180 °C at a rate of 25 °C min^−1^ and maintained for 1 min, followed by an increased to 300 °C at 5 °C min^−1^ and held for 5 min. Injector and detector temperatures were set at 270 °C and 310 °C, respectively. The injection was performed in splitless mode with a 2-µL solution. Helium served as a carrier gas at constant flow rate of 1.5 mL min^−1^, with nitrogen employed as the make-up gas at 29 mL min^−1^. For OCP analysis, the initial column temperature was held at 100 °C for 1 min, then increased to 215 °C at a rate of 10 °C min^−1^, further increased to 285 °C at 4 °C min^−1^, and maintained for 5 min. Injector and detector temperatures were set at 275 °C and 310 °C, respectively. The injection was performed in splitless mode with a 2-µL solution. Helium was used as the carrier gas at constant flow rate of 1.5 mL min^−1^, with nitrogen as the make-up gas at 30 mL min^−1^. A typical GC-ECD chromatogram of PCBs and OCPs is shown in Fig. S1 in the Supplementary material. Each sample was analyzed in triplicate.

The analysis of PAHs (including acenaphthene, acenapththylene, anthracene, benzo[a]anthracene, benzo[a]pyrene, benzo[ghi]perylene, benzo[k]fluoranthene, crysene, dibenzo[a,h]anthracene, fluoranthene, fluorene, indeno[1,2,3-cd]pyrene, naphthalene, phenanthrene and pyrene) was carried out using an Agilent 7890B GC coupled with an Agilent 7000 C series MS/MS triple quadrupole system (Santa Clara, CA, USA) operating in electron-ionization (EI) mode (70 eV). A Sapiens-X5MS (Teknokroma, Sant Cugat del Vallès, Spain) capillary column, consisting of 5% diphenyl 95% dimethyl polysiloxane with dimensions of 20 m × 0.18 mm × 0.36 µm, was employed. The oven temperature was initially set at 60 °C for 1 min, then increased to 190 °C at 15 °C min^−1^, further increased to 265 °C at a rate of 7.5 °C min^−1^, subsequently raised to 310 °C at 3 °C min^−1^, and held for 2 min. The injector temperature was set at 290 °C and the injector was operated in splitless mode with a 1-µL solution. The gas flow rate was adjusted to 0.6 mL min^−1^. Multiple reaction monitoring (MRM) was employed as the acquisition mode, with retention times and collision energies optimized for each transition. Each sample was analyzed once. Table S1 provides information on the different transitions for each analyte, along with the energies used for the fragmentation and retention time.

Limit of detection (LOD) and limit of quantification (LOQ) were determined as the mean of triplicate blank measurements plus three and ten times the standard deviation from three blanks, respectively. Recovery was calculated using the same formula as for trace elements. All analyzed samples were spiked with two surrogates at a concentration of 10 ppb. Depending on the properties of each compound to be quantified, the appropriate surrogate was selected. For PCBs, CB65 was used for the calculation of CB28, CB52, and CB101, while CB166 was employed for CB118, CB153, CB138, and CB180. For OCPs, 2,4,5,6-tetrachloro-m-xylene was used for the quantification of α-HCH, β-HCH, γ-HCH, δ-HCH, heptachlor, aldrin, heptachlor epoxide, endosulfan I, and 4,4′-DDE. Meanwhile, dibutyl chlorendate was used for the quantification of dieldrin, endrin, endosulfan II, 4,4′-DDD, endrin aldehyde, 4,4′-DDT, endosulfan sulfate, and methoxychlor (Fig. S1). For PAHs, five different surrogates were used, with the specific surrogate assigned to each compound detailed in Table S2.

#### Saponins

The MS analyses were carried out using Matrix-assisted Laser Desorption/Ionization (MALDI), performed on a Water QToF Premier mass spectrometer (Waters, Manchester, UK) in the positive ionization mode. The matrix consisted of a mixture of dihydroxyben-zoic acid (DHB, 25 mg) and N,N-dimethylaniline (DMA, 6 µl, 99.9%) in 250 µL of Milli-Q water/acetonitrile (v/v). A matrix solution droplet (1 µL) was placed on a stainless-steel plate and air-dried. 1 µL of the sample solution (1 mg of dried extract in 1 mL of HPLC grade methanol) was then spotted on the top of the matrix crystal and air-dried. The plate was introduced into the MALDI-ToF mass spectrometer. The MALDI source was composed of an Nd-YAG laser with a maximum energy of 104.1 µJ, transferred to the sample in a 2.2 ns pulse (200 Hz). For spectral recording, the quadrupole (rf-only mode) was configured to let the ions pass between m/z 250 and 2000. All the ions were then mass-analyzed using the ToF analyzer (1-s integration time). Mass analyses were performed with the ToF ana-lyzer in reflectron mode, at a FWHM resolution around 10,000. Accurate mass measurements (HRMS) were performed using MALDI-MS(+) with PEG 600–1500 as the external standard (lock mass).

Liquid chromatography analyses were performed with a Waters Acquity UPLC H-Class (Waters, Manchester, UK) composed of a vacuum degasser, a quaternary pump and an autosampler, coupled to a Waters Synapt G2-Si mass spectrometer (Waters, Man-chester, UK). A non-polar column (Acquity UPLC BEH C18; 2.1 × 50 mm; 1.7 µm; Waters) was used at 40 °C. For these analyses, 0.05 mg of saponin extract was dissolved in 1 mL of a Milli-Q water/acetonitrile solution (85/15, v/v). A volume of 5 µL was injected on the column. The gradient was optimized for the compounds in this study and follows a flow rate of 250 µL·min^−1^ of Milli-Q water (with 0.1% formic acid (HCOOH), eluent A), and acetonitrile (CH_3_CN, eluent B). The mobile phase consisted of an elution gradient starting with 85% of eluent A and 15% of eluent B, and reaching 60% of eluent A and 40% eluent B at 6 min, and maintained for 3 min. The ratio was then modified to reach 5% eluent A and 95% eluent B at 11 min, maintained for 1 min and, finally, brought back to 85% eluent A and 15% eluent B at 13 min. This ratio was maintained until the end of the chromatographic run (15 min). Electrospray Ionization (ESI) in positive ionization mode is used for the saponin ion production with typical conditions as follow: capillary voltage 3.1 kV, cone voltage 40 kV, source offset 80 V, source temperature of 120 °C and desolvation temperature of 300 °C (dry nitrogen flow rate 500 L·h^−1^), for a mass range (quadrupole in rf-only mode) between m/z 50 and 2000 (1-s integration time). For the LC-MSMS experiments, precursor ions were mass-selected by the quadrupole and collided against argon (Ar) in the Trap cell of the TriWave device, and the kinetic energy of the laboratory frame (Elab) was selected to afford intense enough product ion signals. The fragment ions were mass-measured with the ToF analyzer.

The relative quantification of saponins within the natural extract was achieved by adding a known quantity (0.1 mg·mL^−1^) of commercially available Hederacoside C (Sigma-Aldrich—Product n° 97,151—M-ClarityTM Program MQ100), a pure saponin from Hedera helix, as internal standard in a solution of saponin extract at a given concentration, typically 0.1 mg·mL^−1^. The spiked solution was analyzed using LC–MS (5-µL injection volume), using the experimental conditions described here above. For each saponin molecule, including Hederacoside C, the corresponding LC–MS ion signals—including all the isotopic compositions—were integrated using the integration algorithm, available under MassLynxTM 4.1 Software. The global ion counts were then used to estimate the Molar Proportions (%) of each saponin within the saponin extracts by assuming similar ionization efficiency for all the saponin congeners (see Table 2). By using the Hederacoside C signal integration as an internal standard and the global yield of extraction, that were determined at 0.045% ± 0.015 and 0.05% for *A. rubens* and *M. glacialis* (note that any Standard deviation was accessible for *M. glacialis* due to the analysis of only one sample), the saponin mass fractions in the animal samples were further calculated and expressed in mg·kg^−1^ (see Table 2).

#### Marine toxins

Lipophilic marine toxins (LMT) were analyzed via LC–MS/MS according to the EU-Harmonised Standard Operating Procedure (SOP) procedure (EURLMB 2015), with the method based on García-Altares et al. 2013. An Agilent 1200 LC (Agilent Inc., Palo Alto, CA, USA) coupled to a 3200 QTRAP mass spectrometer (AB Sciex, Concord, ON, Canada) was utilized. Analytical separation occurred on a X-Bridge C8 column (2.1 × 50 mm, 3.5 μm) with a precolumn (2.1 × 10 mm, 3.5 μm) from Waters (Milford, MA, USA). A binary gradient was employed with water (mobile phase A) and 90/10 (v/v) acetonitrile/water (mobile phase B), both containing 6.7 mM of ammonium hydroxide. Mobile phases were filtrated through 0.2 μm nylon membrane filters (Whatman, Springfield Mill, UK). Chromatographic separations were performed at 30 °C using a flow rate of 500 µL min^−1^. The elution gradient started at 20% B, reached 100% B in 8 min, held for 1 min, then back to 20% B in 1 min and equilibrated for 2 min before the next run started. The injection volume was 10 µL and the auto-sampler was set at 4 °C. A total run time of 12 min was used. LMTs were analyzed in both negative (-ESI) and positive (+ ESI) mode, with two product ions selected per toxin for quantification and confirmation.

Amnesic shellfish poisoning (ASP) toxins were analyzed by LC-UV according to the EU-Harmonised SOP procedure for determination of domoic acid in shellfish by RP-HPLC using UV detection (EURLMB 2008). For LC-UV analyses, an Alliance LC (Waters) and a X-Bridge C18 column (4.6 × 250 mm, 4.6 μm) with a precolumn (2.1 × 10 mm, 3.5 μm) from Waters (Milford, MA, USA) was used. The mobile phase of acetonitrile/water (15:85, v/v) containing 0.1% formic acid was used. All runs were carried out at 40 °C using a flow rate of 1.2 mL min^−1^. The injection volume was 20 μL, the autosampler was set at 4 °C, and the detection was performed at 242 nm.

Paralytic shellfish poisoning (PSP) toxins were determined using an UPLC Acquity H-Class model and FLR Acquity fluorescence detector (Waters Corporation) described in Cabado et al., (2020). Briefly, a Kinetex C18 4.5 μm, 4.6 × 150 mm column and a XSelect CSH C18 4.5 μm guard column from Phenomenex were used. Chromatography conditions were those outlined in the rapid method by Hatfield et al., (2016). The method acceptability criteria were selected to ensure the performance of the method, according to the International Organization for Standardization (ISO) 17025:2005 standards and the UNE-EN 14526:2017.

In all three analytical methods, calibration curve linearities were confirmed before and after each sample set, with curve correlation coefficients (*r*^2^) exceeding 0.98, and slope deviations below 25% to toxin quantifications. Each sample was analyzed in duplicate.

## Results and discussion

### Trace and major elements

The concentration of trace and major elements, along with the recoveries during the extraction process, are shown in Table 1. Additionally, legislated values for their application in animal feed and soil fertilizers (mg kg^−1^, dw) are provided.

**Table 1:** Concentration of major elements and trace metals in starfish (mean ± SD, n= 3), percentage of recovery ± RSD, and the legislated value

	Starfish samples		Recovery ± RSD (%)	Regulated value			
	*A. rubens*	*M. glacialis*		As feed for animals^a^	As fertilizer for soils^b^		
					Class A	Class B	Class C
Major elements (mg kg^−1^, dw)							
Al	47.3 ± 12.3	18.0 ± 8.53	53 ± 3	-	-	-	-
Ca	140637 ± 51826	224209 ± 13563	91 ± 3	-	-	-	-
Fe	123 ± 36.1	59.3 ± 8.58	92 ± 1	-	-	-	-
K	7336 ± 2746	3554 ± 309	97 ± 2	-	-	-	-
Mg	12036 ± 3276	23811 ± 1172	92 ± 1	-	-	-	-
Na	19125 ± 7636	20770 ± 1395	103 ± 2	-	-	-	-
P	4346 ± 1888	1735 ± 187	90 ± 1	-	-	-	-
Sr	801 ± 294	1182 ± 76.5	93 ± 3	-	-	-	-
Trace metals (mg kg^−1^, dw)							
As	12.6 ± 3.05	5.83 ± 0.50	89 ± 1	20	-	-	-
Cd	0.80 ± 0.38	1.12 ± 0.04	55 ± 1	2	0.7	2	3
Co	0.20 ± 0.09	0.07 ± 0.01	96 ± 2	-	-	-	-
Cr	5.25 ± 5.00	1.27 ± 0.25	100 ± 8	-	70	250	300
Cu	5.09 ± 1.93	4.05 ± 0.48	90 ± 1	-	70	300	400
Hg	0.19 ± 0.10	0.10 ± 0.01	100 ± 1	0.5	0.4	1.5	2.5
Mn	5.53 ± 1.95	6.29 ± 0.27	94 ± 4	-	-	-	-
Ni	1.38 ± 1.26	0.55 ± 0.16	81 ± 4	-	25	90	100
Pb	1.34 ± 1.09	0.61 ± 0.05	100 ± 4	10	45	150	200
Zn	92.0 ± 29.7	35.1 ± 1.60	81 ± 1	-	200	500	100

^a^RD 465/2003 on undesirable substances and products in animal nutrition (mg kg^−1^, with a moisture content of 12%)

^b^RD 506/2013 about fertilizer products (in mg kg^−1^, dw)

Regarding the extraction process, recoveries for major elements ranged between 91 and 103%, with the exception of aluminum (53%) being the lowest recovery value. For trace elements, recoveries ranged from 81 to 100%, except for cadmium (55%) which was observed lower. Consequently, the extraction procedure used in this study for the analysis of trace elements in starfish is suitable, as the analyzed elements were within the acceptable range of 80–120%. In addition, precision was determined as the absolute value of the coefficient variation, which was acceptable for all the analyzed compounds (RSD < 10%). However, for Al and Cd, further optimization of the extraction process is recommended. Detection limits for the analyzed elements ranged between 0.01 and 0.2 ng g^−1^.

Two regulations were identified to regulate the concentrations of trace elements. RD 465/2003 regulates the levels of arsenic, cadmium, mercury, and lead in raw materials intended for animal nutrition. None of these concentrations exceeded the permissible limit. In addition, RD 506/2013 regulates the levels of trace elements in fertilizers, specifically cadmium, chromium, copper, mercury, nickel, lead, and zinc. It categorizes these elements into three classes based on their concentrations. Presently, starfish samples would be included in class B due to the cadmium levels, which surpass the maximum concentration of Cd allowed for class A.

When comparing these results with studies in other regions, such as the research performed in the Canary Islands, which evaluated trace element on five echinoderm species, values in a similar concentration range were found (González-Delgado et al. 2024). Metal concentrations were converted from dry weight (dw) to wet weight (ww) using the humidity calculated before and after lyophilization. For asteroid species, the concentrations were comparable for Cd (0.295 vs. 0.34 mg/kg ww) but lower for Zn (28.1 vs. 22.56 mg/kg ww), Pb (0.89 vs. 0.34 mg/kg ww), Cu (3.31 vs. 1.32 mg/kg ww), Ni (1.53 vs. 0.33 mg/kg ww), Cr (1.36 vs. 1.26 mg/kg ww), and Fe (70.3 vs. 32.0 mg/kg ww). Additionally, a study carried out in India analyzed various mollusks, including two starfish species (*Astropectin* sp. and *Pentaceraster* sp.) (Anagha et al. 2022). In contrast to the previous study, lower concentrations were observed for Cd (< LOD vs. 0.80 mg/kg dw), Cr (0.26 vs. 1.27 mg/kg dw), Cu (2.5 vs. 4.05 mg/kg dw), and Zn (28.2 vs. 35. 1 mg/kg dw). However, higher concentrations were reported for Pb (11.9 vs. 1.34 mg/kg dw).

It is noteworthy to consider the elevated calcium levels in starfish samples, even though current regulations do not address it. Several decades ago, limited studies evaluated starfish as a feedstuff, concluding that high calcium content could potentially reduce the growth and protein digestibility in poultry, depending on the feed composition (Stutz and Matterson 1964), although further research should be done to assess the impact in different animal species. Additionally, the content of strontium is also higher in starfish, which can be attributed to their shell and other hard tissues, which correlated with the high calcium concentration. Variations in strontium accumulations may be linked to the species’ ability to accumulate strontium from seawater (Eisler 2010). Riley and Segar (1971) found the highest concentration of Sr in starfish oral skin to be 600 mg kg^−1^ dw (Riley and Segar 1971), while Ohde and Kitano (1984) reported even higher Sr concentrations in various starfish species (1400–1500 mg kg^−1^ dw) (Ohde and Kitano 1984).

### Persistent organic compounds

In 1979, the Stockholm Convention was established to regulate 12 POPs known as “the dirty POPs”, leading to the enactment of Regulation no 850/2004 concerning persistent organic contaminants. The primary objective of this regulation was to safeguard human health and the environment from adverse effects of these contaminants. Subsequently, an additional 16 POPs were also regulated, and with subsequent revisions, Directive 2019/1021 was introduced the newly identified POPs. Under this directive, several POPs such as DDT, HCH (including lindane), dieldrin, endrin, and aldrin were banned due to their harmful effects. Furthermore, the directive-imposed restrictions on the use of PAHs and PCBs. For PCBs, the Directive identified 6 indicator components (CB28, 52, 153, 138 and 180), while for PAHs, it specified 4 indicator values (benzo[a]pyrene (bz[a]Pyr), benzo[a]anthracene (bz[a]Ant), benzo[b]fluoranthene (bz[b]Fla) and crysene (Chr)). These measures aimed to mitigate the risk posed by these hazardous substances to human health and the environment.

Since standardized protocols for the analysis of persistent organic pollutants in starfish matrices were not available, various extraction methods were explored based on published studies with similar matrices. The effectiveness of these procedures was assessed by calculating recoveries, LODs, and LOQs, which are presented in Table S2. Recoveries within the range of 60–115% were considered acceptable, following the guidelines proposed by (Taverniers et al. 2004). Accordingly, the extraction procedures for PCB (63–115%) and OCP (94–116%) were suitable for starfish matrices. However, recoveries for PAH extraction fell below the acceptable range (36–68%). Therefore, it is essential to optimize the extraction procedure for PAH analysis to achieve acceptable recovery percentages and establish a standardized protocol for starfish samples. In terms of detection limits, LODs for PCBs, OPCs and PAHs were between 0.003 and 0.214 ng g^−1^ ww, between 0.008 and 0.940 ng g^−1^ dw, and between 0.001 and 0.098 ng g^−1^ ww, respectively.

**Fig. 1:**
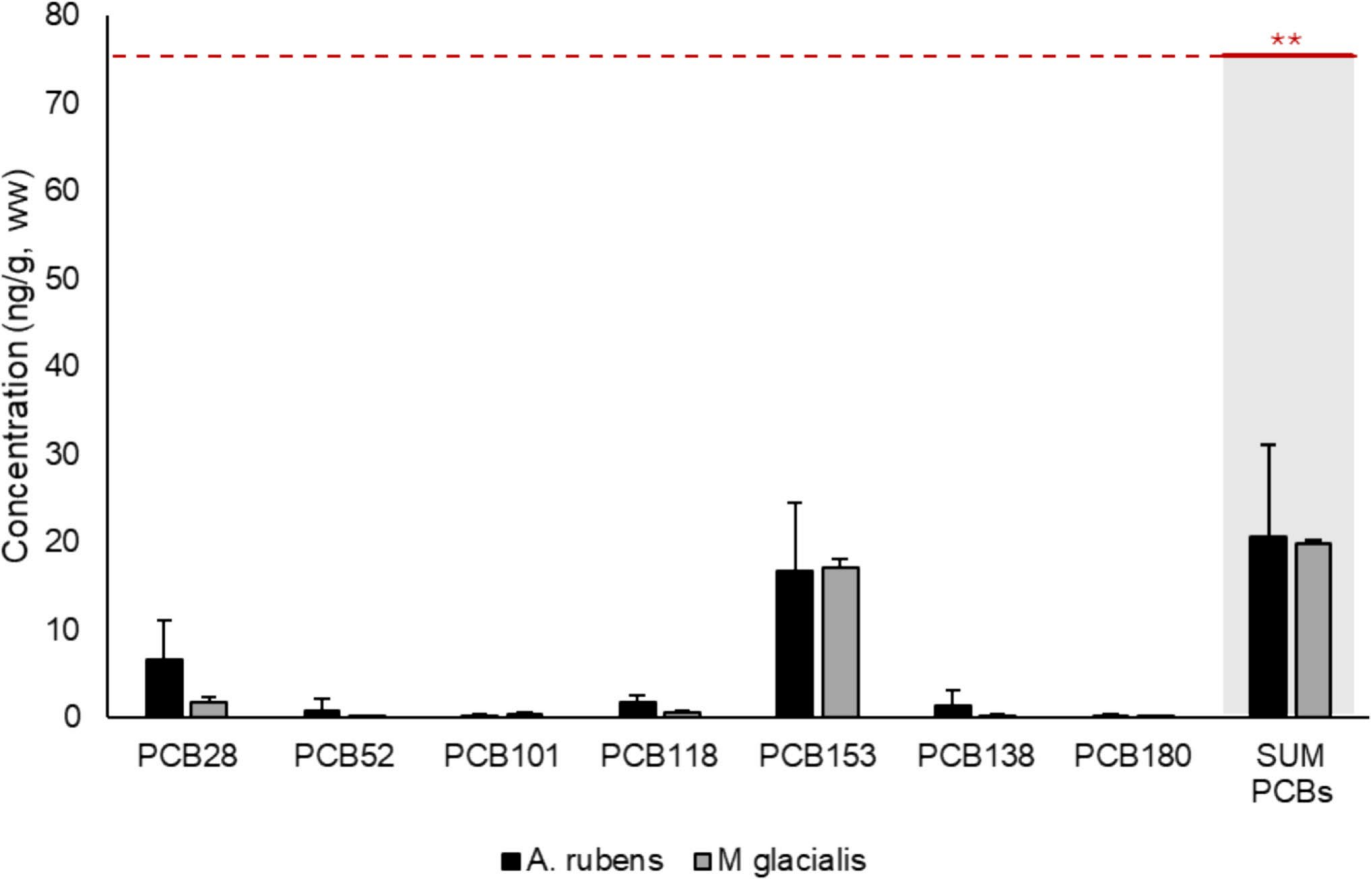
Concentration of PCBs in starfish samples and their regulated values. ^*^Values below the limit of detection. ^**^ Commission Regulation (EC) nº1259/2011 modified from Regulation (EC) nº1881/2006 regarding maximum levels for dioxins, dioxin-like PCBs and non-dioxin-like PCBs in food products (EU, 2011a)

Concentrations of PCBs measured in starfish samples (ng g^−1^ ww), as well as the maximum levels based on the Regulation no 1881/2006 (and its modifications) for foodstuff, are plotted in Fig. 1. To facilitate comparison of these results, PCB concentrations were converted from dry weight (dw) to wet weight (ww) using the humidity content calculated based on the weight measurements taken before and after lyophilization. As can be seen, the highest concentrations of PCBs were attributed to CB153, with values of 16.67 ng g^−1^ ww and 17.12 ng g^−1^ ww observed in both *A. rubens* and *M. glacialis*, respectively. The total concentration of all the PCBs detected in starfish did not exceed the maximum limit set for food products (75 ng g^−1^ ww) (EU, 2011a). No specific legislation which regulates the concentration of PCBs, as well as the case of OCPs and PAHs, in agricultural fertilizers was identified.

The presence of this PCB has been linked to regions characterized by high levels of industrialization (de Boer et al. 2001). The total PCB concentrations detected in this study are comparable to those reported in the Belgian North Sea (Voorspoels et al. 2004), and higher in comparison with the starfish from Antarctica (Goutte et al. 2013).

**Fig. 2:**
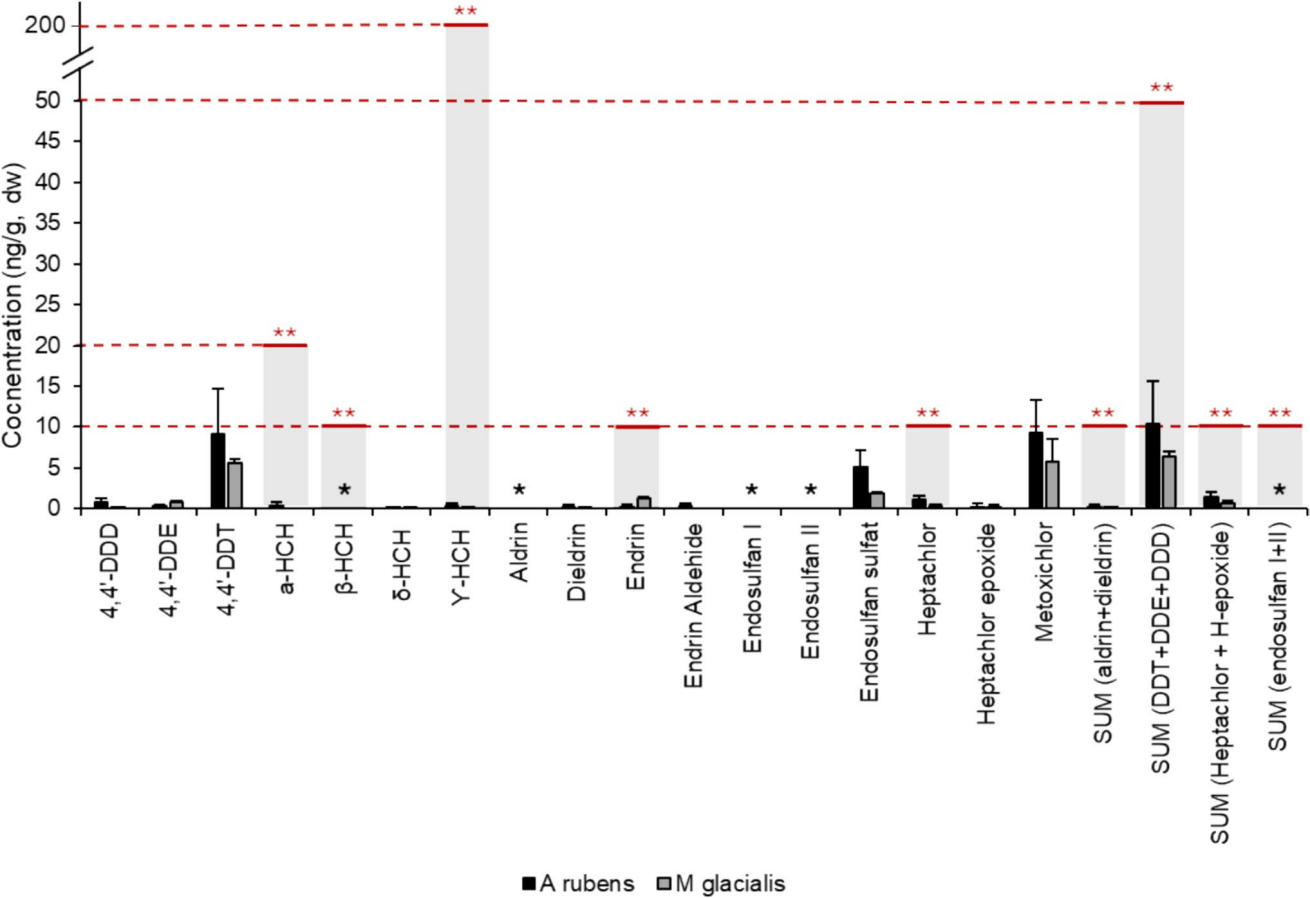
Concentration of OCPs in starfish samples and their regulated values. ^*^Values below the limit of detection. ^**^RD nº465/2003 on undesirable substances and products in animal nutrition (BOE, 2003)

Figure 2 shows OCP concentrations in starfish (ng g^−1^ dw) and the legislation found for the regulation of these contaminants in animal nutrition. Methoxychlor was found to be the predominant OCP in both starfish species, with concentrations of 9.33 and 5.68 ng g^−1^ dw, followed by DDT (9.13 and 5.58 ng g^−1^ dw), and endosulfan sulphate (5.17 and 1.83 ng g^−1^ dw) for *A. rubens* and *M. glacialis*, respectively. However, none of the concentrations of OCPs found in starfish exceeded the current legislation for use as raw material in animal nutrition products (BOE 2003).

The presence of methoxychlor could be attributed to its use as a substitute for DDT, which was banned (Picard et al. 2003). Furthermore, the presence of endosulfan sulphate could be explained because it is a byproduct of the insecticide endosulfan, and finding it in higher proportions compared to endosulfan I suggests that the application of endosulfan has not been a recent development in the field (Commendatore et al. 2015). Comparable concentrations of methoxychlor were reported in mussels from Galicia (Piñeiro et al. 1995), while lower levels of endosulfan sulphate were detected in Morocco (Agnaou et al. 2017). Values of starfish from Belgian North Sea were also comparable to this study (Voorspoels et al. 2004).

**Fig. 3:**
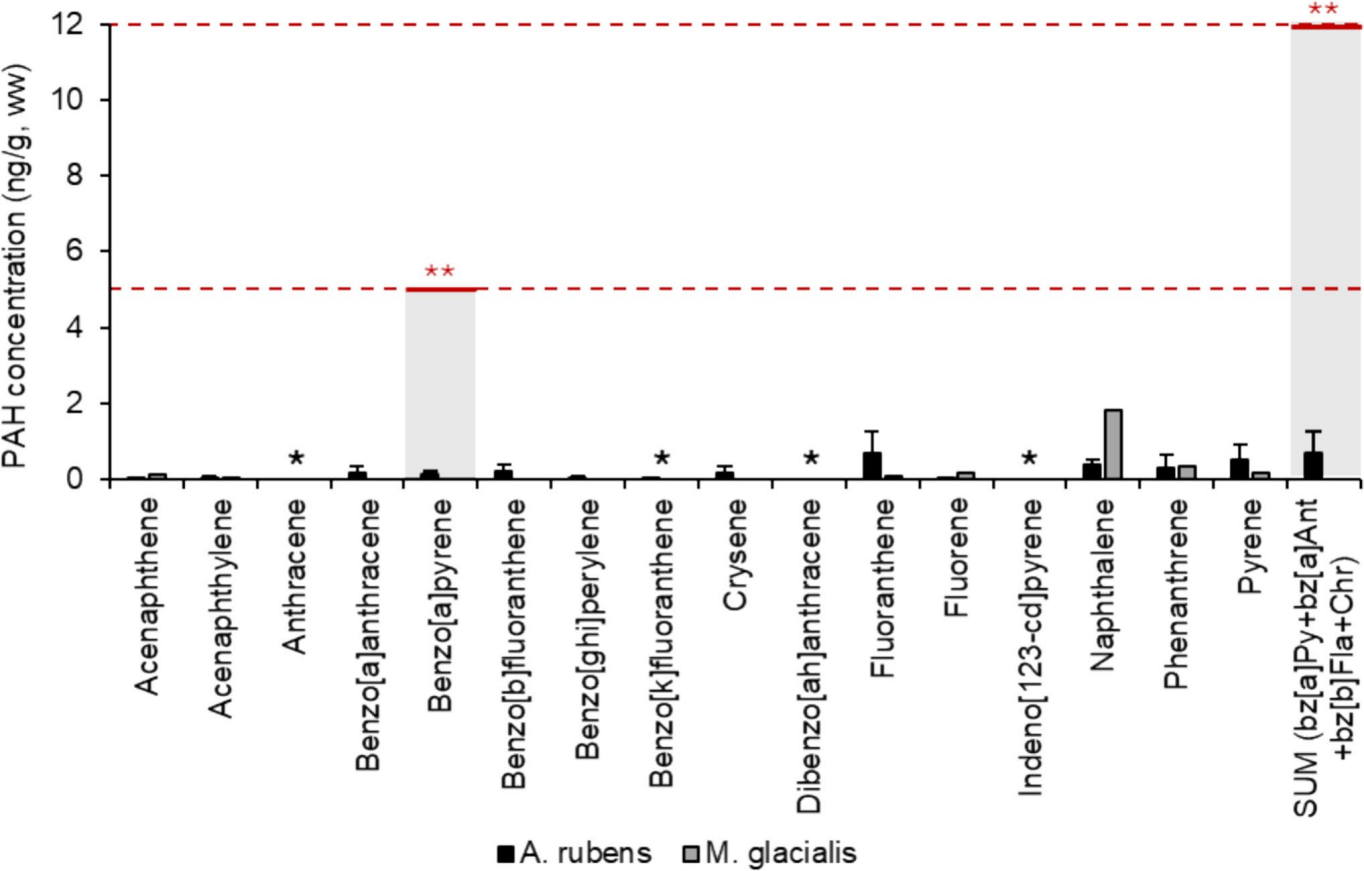
Concentration of PAHs in starfish samples and their regulated values. ^*^Values below the limit of detection. ^**^Regulation (EC) nº835/2011 modified from Regulation (EC) nº1881/2006 as regards the maximum content of polycyclic aromatic hydrocarbons in foodstuffs (EU, 2011b)

In terms of PAHs, in Regulation no 835/2011, the maximum value of benzo[a]pyrene for “unsmoked fish meat” is determined as an indicator of potential environmental contamination. However, it has been observed that PAHs are rapidly metabolized in fresh fish, preventing their accumulation in fish meat. Consequently, maintaining a maximum PAH content in fresh fish may no longer be relevant, and the regulated values in this law are considered for “smoked fish meat”. In Fig. 3, the concentrations of PAHs found in starfish samples (ng g^−1^ ww) are shown and, in addition, the maximum levels determined by this regulation. Nevertheless, none of the concentrations observed in this study exceed the legislation for food products, including benzo[a]pyrene (5 ng g^−1^ ww) as well as for the sum of benzo[a]pyrene, benzo[a]anthracene, benzo[b]fluoranthene and crysene (12 ng g^−1^ ww) (EU, 2011b). If these results were compared with those from a study conducted in South Korea, the concentrations are much lower (Kim et al. 2014). However, similar values have been found in starfish from the Chukchi Sea (Ma et al. 2020).

### Saponins

Saponins are natural glycosides found in plants and some marine animals, such as sea cucumbers and starfish. Saponins are composed of sugar components and a hydrophobic aglycone (Sharmin et al. 2021). Asterosaponins, which are characteristic of starfish, are marine polar steroidal aglycones, characterized by ∆^9(11)^− 3β,6α-dioxygenated steroids with a sulfate group attached at C- 3 and an oligosaccharide chain containing five or six sugar units at C- 6 (Sandvoss et al. 2000; Stonik et al. 2020). In addition, secondary metabolites including steroidal glycosides and polyhydroxy steroids were also reported (Dong et al. 2011). Asterosaponins exhibit a diverse range of biological activities, including cytotoxic, hemolytic, antibacterial, anti-inflammatory, antitumor and cancer-preventing effects (Demeyer et al. 2015; Lazzara et al. 2019; Stonik et al. 2020). Table 2 summarizes the MS determined data for the detected saponins, including their retention times, compositions, *m/z* ratios, and concentrations in the samples. Saponin profiles vary between starfish species. In this study, focusing on two different species, *A. rubens* and *M. glacialis*, distinct types of saponins were identified. The identification of these saponins was based on the literature data, i.e. asterosaponins in *A. rubens* (Demeyer et al. 2015, 2014), and marthasteroside in *M. glacialis* (Dini et al. 1983), by considering the accurate mass measurement data (giving access to the saponin elemental composition) and the relative retention times (in the case of isomeric saponins).

**Table 2:** Compositions and mass error (Δ) from mass spectrometry analysis of the *A.* *rubens* and *M. glacialis* saponin extracts

Saponin	Composition	RT (min)	[M-H]^-^ *m/z*_*HRMS*_ *(Δ ppm)*	*A. rubens* Molar Proportions *(%)*	*A. rubens* Mass Fractions (mg kg^−1^)	*M. glacialis* Molar Proportions *(%)*	*M. glacialis* Mass Fractions (mg kg^−1^)
Unknown- 1	C_58_H_92_O_24_S	11.65	1203.5672 (4.2)	1.78 ± 2.64	5.57 ± 7.73	-	-
Unknown- 2	C_58_H_94_O_24_S	12.19	1205.5836 (4.8)	7.07 ± 4.70	22.8 ± 13.3	-	-
Ruberoside E (^a^) Solasteroside A (^a^)	C_56_H_92_O_26_S	10.63	1211.5553 (2.8)	0.89 ± 1.30	2.85 ± 3.89	-	-
	C_56_H_92_O_26_S	10.48	1211.5519 (2.8)	0.70 ± 1.31	2.51 ± 3.39	-	-
Unknown- 3	C_58_H_94_O_25_S	10.09	1221.5775 (4.0)	5.19 ± 4.27	17.3 ± 13.5	-	-
Ruberoside D	C_57_H_94_O_26_S	11.31	1225.5681 (0.4)	3.07 ± 1.22	10.5 ± 3.90	-	-
Regularoside B	C_57_H_96_O_26_S	9.57	1227.5838 (0.4)	2.14 ± 1.12	7.66 ± 4.78	-	-
Unknown- 4	C_57_H_88_O_27_S	11.24	1235.5175 (1.6)	1.78 ± 1.45	5.82 ± 4.42	-	-
Unknown- 5	C_57_H_90_O_27_S	8.94	1237.5340 (2.2)	2.35 ± 2.78	8.43 ± 10.7	-	-
Ruberoside G	C_57_H_92_O_27_S	11.06	1239.5499 (2.4)	4.67 ± 0.96	16.1 ± 3.51	-	-
Ruberoside B (^b^)	C_57_H_94_O_27_S	10.97	1241.5634 (0.7)	6.42 ± 4.56	22.4 ± 15.6	-	-
Saponin B (^b^)	C_57_H_94_O_27_S	11.53	1241.563 (0.7)	15.6 ± 4.15	53.9 ± 15.7	-	-
Ruberoside A	C_57_H_96_O_27_S	8.34	1243.5796 (1.2)	18.6 ± 6.11	64.3 ± 24.8	-	-
Ruberoside F	C_57_H_94_O_287_S	8.61	1257.5592 (1.4)	11.9 ± 3.47	42.2 ± 16.9	-	-
Saponin C	C_58_H_96_O_28_S	10.11	1271.5736 (0.5)	1.79 ± 1.2	6.23 ± 3.90	-	-
Asteriidoside C	C_623_H_1024_O_31_S	9.54	1373.6096 (3.6)	5.49 ± 3.78	19.4 ± 17.9	-	-
Ruberoside C	C_62_H_104_O_31_S	11.11	1375.6234 (2.2)	2.13 ± 1.06	7.66 ± 5.54	-	-
Asteriidoside B	C_62_H_102_O_31_S	10.94	1387.6241 (2.7)	5.30 ± 1.21	18.7 ± 7.07	-	-
Forbeside A	C_62_H_102_O_33_S	6.04	1405.5980 (2.4)	3.56 ± 3.45	13.3 ± 18.7	-	-
Unknown- 6	C_56_H_92_O_27_S	8.88	1227.5486 (1.4)	-	-	7.39	30.7
Unknown- 7	C_56_H_92_O_27_S	9.99	1227.5486 (1.4)	-	-	4.05	16.8
Marthasteroside B	C_57_H_92_O_27_S	9.20	1239.5469 (1.0)	-	-	21.41	89.0
Marthasteroside C	C_57_H_94_O_27_S	10.67	1241.5625 (0.2)	-	-	49.9	207
Unknown- 8	C_57_H_94_O_28_S	10.00	1257.5579 (0.4)	-	-	1.57	6.52
Unknown- 9	C_57_H_94_O_29_S	7.98	1273.5586 (4.9)	-	-	1.20	4.97
Marthasteroside A_2_	C_62_H_102_O_31_S	8.78	1373.6057 (0.7)	-	-	11.3	46.9
Marthasteroside A_1_	C_62_H_102_O_32_S	8.22	1389.5996 (2.8)	-	-	3.22	13.4

(^a^) Ruberoside E and Solastaroside A are diastereoisomeric saponins and cannot be distinguished by the MS methods used in the present study. (^b^) Ruberoside B and Saponin B are isomeric saponins and are distinguished based on their relative retention times and literature data (Demeyer et al., 2014)

In the case of saponins from *A. rubens*, the predominant asterosaponins were identified as ruberosides A-F (Sandvoss et al. 2003, 2000). As indicated in Table 2, the highest concentration of asterosaponins was observed for ruberoside A (64.25 mg kg^−1^, ww), despite lacking detected biological activity (Jungblut et al. 2020). More striking in the context of the study, is the detection in high quantity of forbeside A (13.3 mg kg^−1^, ww) that is known to exhibit anti-inflammatory and antiviral properties (Findlay et al. 1987). Asteriidoside B (18.7 mg kg^−1^, ww) and C (19.4 mg kg^−1^, ww) were also detected in good yield and are described as displaying moderate cytotoxicity against human lung carcinoma cells (De Marino et al. 1998). Regularoside B (22.4 mg kg^−1^, ww), previously isolated from *Culcita novaeguineae*, was also largely detected and was demonstrated to present a biological activity.

For *M. glacialis*, the predominant detected saponins were marthasteroside C (207 mg kg^−1^, ww) and marthasteroside B (89.0 mg kg^−1^, ww). Limited information is available regarding the biological activity of these asterosaponins. Steroids like dihydromarthasterone were shown to exhibit toxicity to fish and mollusks, while possessing anti-inflammatory, antitumoral and hemolytic properties. In fact, some are used by the Japanese people to eradicate fly larvae due to their surfactant properties (Kijjoa and Sawangwong 2004). In addition, potent anti-inflammatory activity of marthasteroside B was demonstrated, comparable to the positive control from the starfish *Astropecten monacanthus* (Thao et al. 2013). Furthermore, marthasterone and dihydromarthasterone displayed hemolytic activity against sheep blood cells, isolated from *Displasterias brucei* (Mackie et al. 1977). Marthasteroside A_1_ and A_2_ were detected in smaller proportions compared to B and C. Marthasteroside A_1_ and A_2_ are composed of six sugar units and both contain the aglycone thornastrol A (Dini et al. 1983). Tang et al. (2006) reported the moderate cytotoxicity of marthasteroside A_1_ against human leukemia K- 562 cells and human heptoma BEL- 7402 cells (Tang et al. 2006). Unfortunately, no biological activity for marthasteroside A_2_ was identified in the current literature. Conversely, several studies did not attribute specific biological activities to marthasterosides A_1_ and A_2_ (De Marino et al. 1998; Itakura and Komori 1986; Riccio et al. 1986, 1985).

Direct comparison with literature data on the mass proportions of saponins in animal samples is challenging due to the wide diversity in extraction and analysis protocols. For instance, an average saponin content of 50 mg g^−1^ in the dry extract of *Echaniaster sepositus* has been reported in the literature using MS-based methods (Dahmoune et al. 2021). In contrast, approximately 600 mg kg^−1^ of asterosaponins in the wet extract were detected in the stomach of the starfish *L. fusca* (Popov et al. 2019).

Indeed, the absence of current regulations for saponins highlights a significant gap in our understanding of these compounds and their potential impacts. Similarly, there is limited knowledge available regarding asterosaponins across the diverse range of starfish species. Nevertheless, it is crucial that future research endeavors aim to elucidate both positive and negative effects of these compounds on animals and agriculture.

### Marine toxins

The production of marine toxins by phytoplankton proliferation is a well-known phenomenon, with approximately 100 microalgal species capable to produce toxins which can cause intoxication in both animals and humans. As mentioned, starfish are among the primary predators of marine shellfish such as mussels, clams, and razor clams. These filter-feeding mollusks have the ability to accumulate marine toxins in their bodies through the filtration of toxic microalga (Farabegoli et al. 2018). Therefore, quantifying marine toxins in starfish samples is essential due to their potential to bioaccumulate these toxins from their diet.

In this study, standardized procedures were employed (see materials and methods), and starfish samples were analyzed in an accredited laboratory. The concentrations of LMTs, PSP and ASP toxins in starfish samples, as well as the regulated limits, were summarized in Table S3. In all cases, the measured values were found to be below LOQ. Furthermore, these values were also found to be below the regulated limits set by Regulation no 853/2004, as amended by Regulation no 2013/786 and Regulation no 2021/1374, for bivalve in all cases (EU 2021, 2013; Visciano et al. 2016).

## Conclusions

The decline in shellfish production along the Galician coast, attributed to the increasing starfish population, poses a significant challenge. While governmental authorization for starfish culling exists, the subsequent management of resulting waste remains a topic of debate. Proposing a circular economy approach, there is an opportunity to valorize starfish waste by transforming it into a value-added product. However, any product derived from starfish must adhere to EU regulations governing contaminant levels, ensuring suitability for use as animal feed or agricultural fertilizer.

For this reason, a comprehensive analysis was performed on the starfish matrix using optimized analytical methodologies to assess trace elements (both major and trace metals), POPs such as PCBs, OCPs, and PAHs, as well as marine toxins and saponins. For the analysis of metals, recoveries between 81 and 103% were achieved, apart from aluminum and cadmium with 53% and 55%, respectively. While acceptable recoveries were determined for PCBs (63–115%) and OCPs (94–116%), further optimization of the PAH analysis protocol may be done. Notably, the absence of EU regulations for saponins underscores the need for continued research in this area. Recoveries for saponins ranged from 76 to 83%, demonstrating the efficacy of the novel analytical procedures employed.

The results obtained for these pollutants were cross-referenced with existing legislation. Both starfish species could potentially serve as animal feed or be used in agriculture, since the content of the selected pollutants does not exceed any of the EU regulated limits. This presents an opportunity to repurpose starfish, which are currently regarded as waste. To evaluate their potential impacts on agriculture, such as crop productivity, and as animal feed, particularly for fish, ongoing studies have been initiated, considering the variability among species.

## Supplementary information

Below is the link to the electronic supplementary material.

ESM 1 (DOCX 69.2 KB)

## Data Availability

All authors declare that all data and materials as well as software application or custom code support their published claims and comply with field standards.
