# Supplementary material for: Determining toxins and harmful contaminants in starfish for future application as organic fertilizer and animal feed
Source: Environ Sci Pollut Res Int. 2025 May 7;32(20):12221–35. doi: 10.1007/s11356-025-36430-3 (PMC12098462; doi:10.1007/s11356-025-36430-3)
Supplement: Supplementary file 1 — (DOCX 69.2 KB) [file 11356_2025_36430_MOESM1_ESM.docx]

**SUPPLEMENTARY MATERIAL FOR THE ARTICLE**

**Determining toxins and harmful contaminants in starfish for future application as organic fertilizer and animal feed**

Marta Turull, Belén Budiño, Philippe Savarino, Pascal Gerbaux, Maria Rambla-Alegre, Santiago Cabaleiro, Sergi Díez

**Figure S1**. (a) GC-ECD chromatogram of PCBs (1: PCB30, IS; 2: PCB204, IS; 3: PCB65, surrogate; 4: PCB166, surrogate; 5: PCB28; 6: PCB52; 7: PCB101; 8: PCB118; 9: PCB138; 10; PCB153; 11: PCB180). (b) GC-ECD chromatogram of OCPs (1: PCB65, IS; 2: PCB166, IS; 3: 2,4,5,6-tetrachloro-m-xylene, surrogate; 4: dibutyl chlorendate, surrogate; 5: α-HCH; 6: β-HCH; 7: ϒ-HCH; 8: δ-HCH; 9: heptachlor; 10: aldrin; 11: heptachlor epoxide; 12: endosulfan I; 13: 4,4’-DDE; 14: dieldrin; 15: endrin; 16: endosulfan II; 17: 4,4’-DDD; 18: endrin aldehyde; 19: 4,4’-DDT; 20: endosulfan sulfate; 21: metoxychlor).


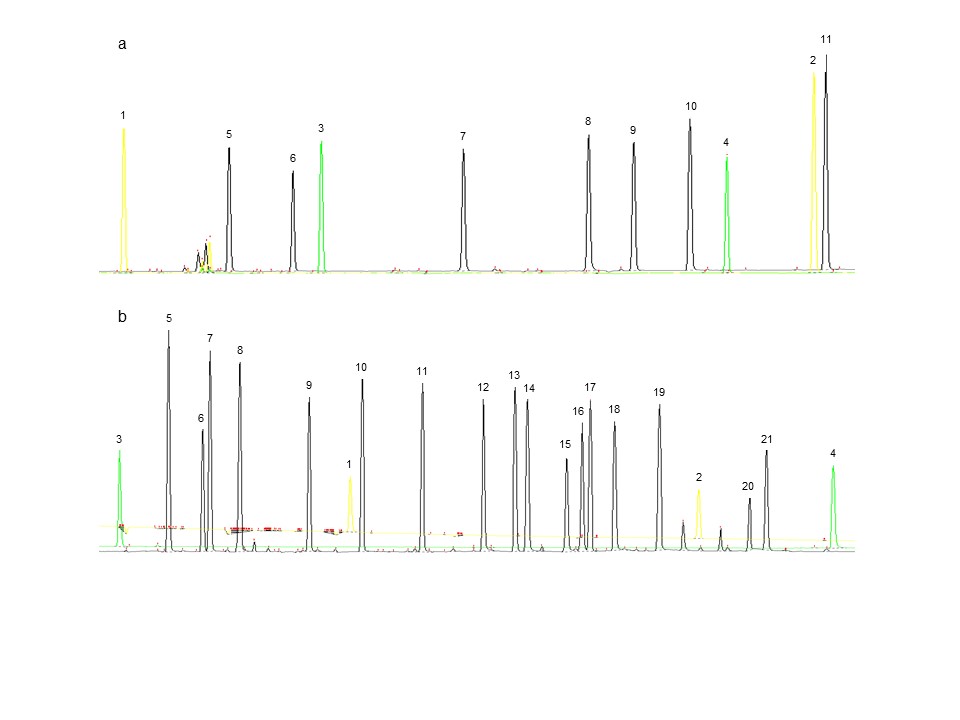


**Table S1.** MRM transitions used for quantifier and qualifiers with collision energies optimized for the analytes and the surrogates.

|  | **RT (min)** | **Transition of quantification** | **Collision energy (eV)** | **Transition of identification** | **Collision energy (eV)** |
| --- | --- | --- | --- | --- | --- |
| Naphthalene-d8 | 6.40 | 136>136 | 22 |  |  |
| Naphthalene | 6.41 | 128>102 | 22 | 128>127 | 20 |
|  |  |  |  |  |  |
| Acenaphthylene | 9.03 | 152>150 | 40 | 152>151 | 40 |
| Acenaphthene | 9.31 | 154>152 | 40 | 153>152 | 40 |
| Fluorene | 10.18 | 166>165 | 30 | 166>163 | 34 |
| Phenanthrene | 12.08 | 178>176 | 34 | 178>152 | 30 |
| Anthracene-d10 | 12.15 | 188>160 | 25 | 94>80 | 10 |
| Anthracene | 12.19 | 178>176 | 34 | 178>152 | 30 |
|  |  |  |  |  |  |
| Fluoranthene | 15.02 | 202>200 | 50 | 202>201 | 50 |
| Pyrene-d10 | 15.56 | 212>208 | 40 | 212>210 | 25 |
| Pyrene | 15.61 | 202>200 | 50 | 202>201 | 30 |
|  |  |  |  |  |  |
| Benzo[a]anthracene-d12 | 19.09 | 240>236 | 35 | 240>238 | 20 |
| Benzo[a]anthracene | 19.15 | 228>226 | 38 | 228>224 | 38 |
| Chrysene | 19.25 | 228>226 | 38 | 228>224 | 38 |
|  |  |  |  |  |  |
| Benzo[b]fluoranthene | 22.61 | 252>250 | 42 | 250>248 | 40 |
| Benzo[k]fluoranthene | 22.71 | 252>250 | 42 | 250>248 | 40 |
| Benzo[a]pyrene | 23.76 | 252>250 | 40 | 250>248 | 40 |
| Perylene-d12 | 23.91 | 264>260 | 40 | 264>236 | 25 |
| Indeno[1,2,3-cd]pyrene | 28.15 | 276>274 | 38 | 138>124 | 38 |
| Dibenzo[a,h]anthracene | 28.33 | 278>276 | 42 | 276>274 | 30 |
| Benzo[ghi]perylene | 29.15 | 276>274 | 42 | 274>272 | 42 |

**Table S2.** Recoveries of PCBs, OCPs and PAHs analysis, limit of detection (LOD) and limit of quantification (LOQ). ww: wet weight, dw: dry weight

|  | **LOD** | **LOQ** | **Recovery (%)** |
| --- | --- | --- | --- |
| ***PCBs (ng g^-1^ ww)*** |  |  |  |
| PCB28 | 0.015* | 0.039* | 115 |
| PCB52 | 0.214* | 0.353* | 115 |
| PCB101 | 0.144* | 0.340* | 115 |
| PCB118 | 0.116* | 0.173* | 63 |
| PCB153 | 0.070* | 0.129* | 63 |
| PCB138 | 0.080* | 0.209* | 63 |
| PCB180 | 0.003* | 0.005* | 63 |
| ***OCPs (ng g^-1^ dw)*** |  |  |  |
| α-HCH | 0.433 | 0.957 | 116 |
| β-HCH | 0.940 | 1.258 | 116 |
| ϒ-HCH (Lindane) | 0.049 | 0.100 | 116 |
| δ-HCH | 0.051 | 0.109 | 116 |
| Heptachlor | 0.008 | 0.014 | 116 |
| Aldrin | 0.644 | 1.554 | 116 |
| Heptachlor epoxide | 0.070 | 0.165 | 116 |
| Endosulfan I | 0.202 | 0.428 | 116 |
| 4,4’-DDE | 0.024 | 0.034 | 116 |
| Dieldrin | 0.059 | 0.080 | 94 |
| Endrin | 0.077 | 0.121 | 94 |
| Endosulfan II | 0.066 | 0.152 | 94 |
| 4,4’-DDD | 0.083 | 0.157 | 94 |
| Endrin aldehide | 0.134 | 0.305 | 94 |
| 4,4’-DDT | 0.052 | 0.065 | 94 |
| Endosulfan sulfat | 0.197 | 0.517 | 94 |
| Metoxichlor | 0.034 | 0.061 | 94 |
| ***PAHs (ng g^-1^ fw)*** |  |  |  |
| Naphthalene | 0.098* | 0.258* | 36 |
| Acenapththylene | 0.001* | 0.003* | 57 |
| Acenaphthene | 0.008* | 0.014* | 57 |
| Fluorene | 0.006* | 0.008* | 57 |
| Phenanthrene | 0.039* | 0.074* | 57 |
| Anthracene | 0.067* | 0.192* | 57 |
| Fluoranthene | 0.005* | 0.020* | 53 |
| Pyrene | 0.006* | 0.013* | 53 |
| Benzo[a]anthracene | 0.009* | 0.020* | 68 |
| Chrysene | 0.002* | 0.004* | 68 |
| Benzo[b]fluoranthene | 0.004* | 0.011* | 55 |
| Benzo[k]fluoranthene | 0.008* | 0.022* | 55 |
| Benzo[a]pyrene | 0.002* | 0.004* | 55 |
| Indeno[1,2,3-cd]pyrene | 0.001* | 0.003* | 55 |
| Dibenzo[a,h]anthracene | 0.001* | 0.001* | 55 |
| Benzo[ghi]perylene | 0.003* | 0.008* | 55 |

*Values of LOD and LOQ calculated from dw to ww with the average of %humidity from samples of starfish

**Table S3.** Content of marine toxins in starfish and their regulation limits.

| **Toxins** | **LOQ** | ***A. rubens*** | ***M. glacialis*** | **Regulated value** |
| --- | --- | --- | --- | --- |
| **Paralytic toxins (PSP)** | | | | |
| **STX**  (µg equiv STX 2HCl Kg^-1^) | 40 | <LOQ | <LOQ | 800^c^ |
| **NEO**  (µg equiv STX 2HCl Kg^-1^) | 150 | <LOQ | <LOQ |  |
| **dcNEO**  (µg equiv STX 2HCl Kg^-1^) | 120 | <LOQ | <LOQ |  |
| **GTX2&3**  (µg equiv STX 2HCl Kg^-1^) | 25 | <LOQ | <LOQ |  |
| **GTX1&4**  (µg equiv STX 2HCl Kg^-1^) | 150 | <LOQ | <LOQ |  |
| **dcGTX2&3**  (µg equiv STX 2HCl Kg^-1^) | 60 | <LOQ | <LOQ |  |
| **dcSTX**  (µg equiv STX 2HCl Kg^-1^) | 40 | <LOQ | <LOQ |  |
| **GTX5**  (µg equiv STX 2HCl Kg^-1^) | 28 | <LOQ | <LOQ |  |
| **C1&C2**  (µg equiv STX 2HCl Kg^-1^) | 20 | <LOQ | <LOQ |  |
| **GTX6**  (µg equiv STX 2HCl Kg^-1^) | 20 | <LOQ | <LOQ |  |
| **C3&C4**  (µg equiv STX 2HCl Kg^-1^) | 34 | <LOQ | <LOQ | - |
| **Amnesic toxins (ASP)** | | | | |
| **Domoic acid**  (mg domoic acid Kg^-1^) | 2 | <LOQ | <LOQ | 20^a^ |
| **Lipophilic marine toxins** | | | | |
| **OA**  (µg equiv OA Kg^-1^) | 40 | <LOQ | <LOQ | 160^c^ |
| **DTX-1**  (µg equiv OA Kg^-1^) | 40 | <LOQ | <LOQ |  |
| **DTX-2**  (µg equiv OA Kg^-1^) | 24 | <LOQ | <LOQ |  |
| **PTX-1**  (µg equiv OA Kg^-1^) | 50 | <LOQ | <LOQ | - |
| **PTX-2**  (µg equiv OA Kg^-1^) | 50 | <LOQ | <LOQ |  |
| **YTX**  (mg equiv YTX Kg^-1^) | 0.1 | <LOQ | <LOQ | 3.75^b^ |
| **45 OH YTX**  (mg equiv YTX Kg^-1^) | 0.1 | <LOQ | <LOQ |  |
| **Homo YTX**  (mg equiv YTX Kg^-1^) | 0.1 | <LOQ | <LOQ |  |
| **45 OH homoYTX**  (mg equiv YTX Kg^-1^) | 0.05 | <LOQ | <LOQ |  |
| **AZA-1**  (µg equiv AZA Kg^-1^) | 30 | <LOQ | <LOQ | 160^a^ |
| **AZA-2**  (µg equiv AZA Kg^-1^) | 54 | <LOQ | <LOQ |  |
| **AZA-3**  (µg equiv AZA Kg^-1^) | 56 | <LOQ | <LOQ |  |
| **SPX-1**  (µg Kg^-1^) | 25 | <LOQ | <LOQ | - |
| **GYM**  (µg Kg^-1^) | 25 | <LOQ | <LOQ | - |
| **PnTX-G**  (µg Kg^-1^) | 2 | nd | nd | - |

^a^Maximum limits for marine biotoxins in Regulation (EC) nº 853/2004 for bivalve mollusks (EC, 2004).

^b^Permited limits of yessotoxins in live bivalve molluscs in Regulation (EU) No 786/2013 (EC, 2013). ^c^Specific hygiene requirements for food of animal origin in the Regulation (EU) 2021/1374 (EC, 2021). Saxitoxin: STX; Neosaxitoxin: NEO; decarbamoylneosaxitoxin: dcNEO; Gonyautoxin 2 y 3: GTX2&3; Gonyautoxin: GTX1&4; decarbamoylgonyautoxin 2 y 3: dcGTX2&3; decarbamoilsaxitoxin: dcSTX; Gonyautoxin 5: GTX5; Gonyautoxin 6: GTX6; Okadaic acid: OA; Dinofisistoxin-1: DTX-1; Dinofisistoxin-2: DTX-2; Yessotoxin: YTX; 45-hydroxy-yesotoxin: 45 OH YTX; 1-homoyessotoxin: homoYTX; 1-a-45-OH-hydroxy-homoyessotoxin (45 OH homoYTX); Azaspiracid-1: AZA-1; Azaspiracid-2: AZA-2; Azaspiracid-3: AZA-3; Pectenotoxin-2: PTX-1; Pectenotoxin-2: PTX-2; 13-desmethylspirolide C: SPX-1; Gymnodimine: GYM; Pinnatoxin-G: PnTX-G; Domoic acid: DA. Nd: non-detected.
